# End-of-Life Expectations and Meaning-Making Among First-Year Medical Residents: A Phenomenological Study in a Mexican General Hospital

**DOI:** 10.3390/bs16101786

**Published:** 2026-10-01

**Authors:** Jaime Isael Flores-Rosas, Osmar Antonio Jaramillo-Morales, Juan Ramón Ruíz-Carlo, Nereida Violeta Vega-Cabrera, José Miguel Torres-Martel, Juan de Dios Recio-Chávez

**Affiliations:** 1 Coordinación Clínica de Cirugía, HGZMF 2, Instituto Mexicano del Seguro Social, Irapuato 36650, Guanajuato, Mexico; zrewolf@gmail.com; 2 Departamento de Enfermería y Obstetricia, División de Ciencias de la Vida, Campus Irapuato-Salamanca, Universidad de Guanajuato, Irapuato 36500, Guanajuato, Mexico; nv.vega@ugto.mx; 3 Coordinación Clínica de Educación e Investigación en Salud, HGZMF 2, Instituto Mexicano del Seguro Social, Irapuato 36650, Guanajuato, Mexico; juan.ruizca@imss.gob.mx; 4 Facultad de Medicina, Universidad Autónoma de Querétaro, Santiago de Querétaro 76176, Querétaro, Mexico; dr.torrescardiologia@gmail.com; 5 Unidad Académica de Filosofía, Universidad Autónoma de Zacatecas, Zacatecas 98000, Zacatecas, Mexico

**Keywords:** death, medical culture, phenomenology, end of life, bioethics, narrative medicine

## Abstract

End of life is a profound and culturally charged human dimension. Bioethics and palliative medicine have promoted a shift from the traditional biomedical model toward person-centered care that preserves dignity through the dying process. Physicians-in-training live in continuous contact with illness and death, yet are rarely invited to reflect on their own finitude. Reflection on one’s own mortality has been linked to greater comfort discussing prognosis, more patient-centered end-of-life decision making, and reduced avoidance behaviors in clinical encounters with dying patients; however, most training programs address death only as a technical or bioethical topic, rarely as a personal experience requiring processing. This study explored the end-of-life expectations and meaning-making of first-year medical residents at a Mexican general hospital, considering the influence of medical culture and sociocultural discourse. A qualitative phenomenological design was used. Data were collected through asynchronous, self-recorded video interviews guided by five open-ended questions addressing illness and death, family, autonomy, medical care at the end of life, advance directives, and euthanasia. Purposive sampling included twenty-eight first-year residents (2024–2025 cohort) who had previously completed coursework in medical ethics, giving the final sample of twenty-eight participants. Data were analyzed using an interpretive phenomenological approach supported by ATLAS.ti. Open coding of experiential meaning units was followed by iterative hermeneutic interpretation. Participants described the end of life through two tensions: an idealized, peaceful death surrounded by family, and a pragmatic view shaped by clinical exposure to suffering and futile treatment. Participants uniformly favored palliative care and expressed a preference for dying at home in the case of a terminal illness. Attitudes toward euthanasia ranged from support as an individual right to religious/ethical opposition, frequently anchored in personal grief experiences. Advance directives were positively valued but rarely formalized. Emotional overflow, including tears, occurred in some interviews. Despite clinical experience, medical residents are not immune to the emotional and existential tensions surrounding death. Findings suggest that integrating reflective, narrative-based pedagogical strategies into residency training may help to process death-related distress and strengthen person-centered, dignity-preserving end-of-life care.

## 1. Introduction

Death is the only certainty of human existence, yet contemporary societies—Mexico among them—continue to treat it as an uncomfortable presence, displaced from everyday conversation and from professional training spaces, including those where future health professionals are formed. Philippe Ariès traced this shift historically, describing how death moved from a domestic, community-witnessed event to an institutionalized, hospital-based process, a transition that stripped dying of much of its earlier social and symbolic meaning (Ariès, 1975/2000). Norbert Elias similarly argued that the loneliness of the dying in modern societies is not accidental but structurally produced, as illness in others generates discomfort and avoidance (Elias, 1982/2009). Byung-Chul Han extends this analysis to what he terms the “palliative society,” in which pain is interpreted as a sign of weakness to be eliminated rather than a meaningful part of existence, and where a culture of unbroken positivity forecloses any real engagement with finitude (Han, 2020/2021).

This avoidance is mirrored and reinforced within medicine itself. The biomedical model, heir to a Cartesian paradigm, has historically construed the human body as a machine and death as a therapeutic failure rather than a natural, even dignified, closure of the life cycle (Foucault, 1963/2004; Mejía Estrada & Romero Zepeda, 2017). Michel Foucault described how the “medical gaze” reorganized the clinical encounter around lesions and objectifiable disease rather than the subjective experience of the person who suffers (Foucault, 1963/2004; Svenaeus, 2023). Although the biopsychosocial model and the World Health Organization’s own definition of health—as complete physical, mental, and social well-being, not merely the absence of disease—call for a person-centered approach (World Health Organization, 1946), hospital culture continues to privilege the prolongation of life over its quality, particularly in Latin American medical settings (Arrubarrena-Aragón, 2011).

Physicians-in-training occupy a singular position within this tension. They are professionals whose identity is still under construction, who face life-and-death decisions daily, and who work within institutional cultures that rarely promote open dialogue about death or about their own fears and expectations (Loayssa-Lara et al., 2009). At the same time, they belong to a generation increasingly exposed, at least nominally, to medical ethics, bioethics, and palliative care coursework intended to cultivate empathy and person-centered practice (Escribano, 2024). On that matter, a systematic scoping review by Ho et al. (2020) synthesized how encounters with death and dying shape the professional “personhood” of medical students, identifying recurring effects across training contexts not only on identity formation, but also on emotional regulation and ethical outlook (Ho et al., 2020). Recognizing one’s own finitude has been proposed as a pathway toward more compassionate, dignity-preserving care (Frankl, 1946/2015; Rohden, 2008), an ethical stance grounded in Kant’s principle of treating persons always as ends in themselves, never merely as means (Rohden, 2008).

Expectations about illness and death are shaped by past experience, prevailing social constructs, and cultural context, and they rarely account for adversity; when reality departs from these expectations, an emotional response follows (Heidegger, 1927/1997; Katz, 1980). This gap between expectation and reality is not only psychological but ontological; Heidegger described death as “the possibility of the impossibility of existing,” a possibility that is one’s own, non-relational, and certain, yet indeterminate (Heidegger, 1927/1997, p. 271). In Mexico, death occupies an ambivalent cultural space: ritually celebrated in traditions such as the Day of the Dead, yet still largely absent from clinical and family conversations, reinforced by fatalistic religious narratives and by strikingly low rates of advance-care planning—fewer than 1% of Mexican adults are estimated to have formalized end-of-life instructions (Vélez, 2026).

Atul Gawande has argued that medical education equips physicians to save lives but rarely teaches them how to accompany dying (Gawande, 2014). This educational gap has measurable consequences; globally, only an estimated 14% of people in need of palliative care actually receive it (World Health Organization, 2020), and in Mexico, structural barriers—including inequitable access to opioid analgesia—compound this gap (Covarrubias-Gómez et al., 2023). A qualitative study among nurses in Mexico found that physicians frequently avoid engaging with dying patients even when death is imminent, underscoring an urgent training need (Marván et al., 2017). Narrative medicine, which recovers individual and collective narratives as a pedagogical tool, has been proposed as a means of shifting the classical medical gaze and supporting clinician well-being (Loy & Kowalsky, 2024).

Despite this growing body of theoretical and empirical evidence, few qualitative studies have explored how physicians-in-training themselves narrate and make sense of their own mortality. The group of first-year residents in medical specialties was selected because it was hypothesized that at this stage, although they are already immersed in the modern ethical-humanistic theoretical approach, they are still less shaped by the entrenched norms of hospital culture, which is why they constitute a strategic group for exploring how professional and sociocultural discourses converge in the construction of meaning around death. This study therefore aimed to explore the narrative representations, expectations, and collective meanings that first-year medical residents construct around the end of life, considering the influence of medical culture, professional socialization, and the sociocultural discourses in which they were formed. Specific objectives were not only to analyze how residents conceptualize their own end of life but to explore discourses surrounding terminal illness, autonomy and euthanasia; and also to understand how medical training shapes advance-care planning and end-of-life decision-making.

## 2. Materials and Methods

### 2.1. Study Design

This study was informed by Heideggerian interpretive phenomenology and by van Manen’s phenomenological orientation toward lived experience. The aim was not only to identify recurrent themes but also to interpret the meanings participants attributed to finitude, suffering, autonomy, and end-of-life decision-making within the context of medical professional socialization.

This interpretive phenomenological orientation was a deliberate methodological choice. A descriptive approach seeks the essential, invariant structure of an experience through transcendental reduction, in which the concrete socio-cultural and professional context of the participant is bracketed as incidental to that structure. Our research questions, however, concern precisely the opposite: how meanings around finitude are shaped by, and inseparable from, medical culture, professional socialization, and sociocultural discourse. Heidegger’s ontology treats being-in-the-world—including one’s professional situatedness—as constitutive of, rather than prior to, meaning; death is disclosed not as an abstract biological endpoint but as interpreted from within a particular mode of be-ing-in-the-world. Van Manen’s phenomenology of practice (Rich et al., 2013; van Manen, 2018) operationalizes this ontology for professional and practice-based lifeworlds, making it particularly suited to populations—such as physicians-in-training—whose relation to death is inextricable from clinical practice and professional identity formation. Following recent comparative guidance on selecting among empirical phenomenological methodologies (Öhlén & Friberg, 2023) and on aligning the research question with its underlying philosophical commitments (Tavakol & Sandars, 2025), we therefore adopted the Heideggerian/van Manen interpretive tradition instead of a descriptive alternative, since our aim was to interpret situated meaning within medical culture rather than to isolate an essence of the experience abstracted from it.

Four concrete operations structured data generation and analysis in line with this framework. First, rather than bracketing the principal investigator’s clinical and educational pre-understanding, this was made explicit through the reflexive account in Section 2.6 and revisited throughout coding, consistent with the hermeneutic (rather than tran-scendental) variant of phenomenological reduction. Second, open codes were examined for their relation to van Manen’s four lifeworld existentials—lived body, lived time, lived space, and lived relation—for example, codes describing dying ‘surrounded by family’ were read as expressions of lived relation, and codes describing ‘suffering avoidance’ and ‘dignity’ as expressions of lived body. Third, analysis proceeded through a hermeneutic circle in which individual narrative units were interpreted in light of the emerging categories and, reciprocally, the categories were revised in light of individual accounts, rather than a single linear coding pass. Fourth, the interpretive claims presented in the Results and Discussion were developed through iterative phenomenological writing and rewriting, in which draft interpretations were repeatedly checked against, and revised in response to, the verbatim transcripts, consistent with van Manen’s treatment of writing as itself an analytic act.

### 2.2. Setting and Participants

The study was conducted between July 2025 and November 2025 at the Hospital General de Zona con Medicina Familiar No. 2 (HGZ MF No. 2) of the Instituto Mexicano del Seguro Social (IMSS), in Irapuato, Guanajuato, Mexico. Purposive (non-probabilistic) sampling was used to recruit first-year medical residents from the 2024–2025 cohort. Inclusion criteria were: (1) enrollment in the first year of a medical specialty at HGZ MF No. 2 during 2024–2025; (2) prior completion of coursework in medical ethics and professionalism; and (3) written informed consent. Exclusion criteria were: (1) residents from generations other than the 2024–2025 cohort, (2) residents on medical leave during the collection period. Non-participation criteria: (1) refusal to participate or inability to complete the interview for reasons beyond the participant’s control; (2) failure to respond to the interview questions.

Recruitment was conducted through a synchronous online brief meeting via video-mediated communication, rather than a traditional face-to-face interaction. Twenty-eight residents were initially recruited (17 women, 11 men; age range 25–33 years), representing family medicine, anesthesiology, surgery, internal medicine, and pediatrics.

Rather than relying on thematic or data saturation as the criterion of sample adequacy, an intentional census sampling was carried out. All 28 first-year resident doctors of the 2024–2025 cohort at HGZ MF No. 2 who had previously completed coursework in medical ethics and met the inclusion criteria were included. The criterion of theoretical saturation was not applied since it was a finite and accessible population. The sample sufficiency was supported by Malterud et al.’s (2016) Information Power model (Malterud et al., 2016), considering: (a) a well-defined study objective, (b) a highly specific sample, (c) use of established theories as an interpretative framework, (d) quality of dialogue, and (e) cross-case analysis strategy. Based on these criteria, the census sample (28) is sufficient.

The target of 28 participants was set in the protocol design, acknowledging the possibility that some residents might not participate. Because the population of interest was itself delimited by institutional and curricular criteria, sufficiency was defined by population coverage rather than by the point at which no new codes emerged during concurrent analysis.

Although thematic saturation was not used as a formal stopping criterion, substantial convergence was observed across participants regarding preferences for palliative care, home-based end-of-life care, respect for autonomy, and concerns regarding disproportionate treatment. This convergence reinforced the adequacy of the final sample for phenomenological interpretation.

### 2.3. Data Collection

Data were collected through individual, asynchronous, self-recorded video interviews, a modality consciously selected to reduce social-desirability bias toward a physician-investigator and to allow reflection without the time pressure of clinical duties; a recognized limitation of this approach is the absence of real-time probing and of observable nonverbal cues during the interview itself, which invariably limits the phenomenological depth. Participants received a written guide containing five open-ended questions, derived from the theoretical framework, designed by the investigator to prompt deep reflection on personal mortality, the role of family, autonomy, end-of-life medical care, advance directives, and euthanasia (Appendix A). The initial version of the guide was reviewed by two subject matter experts, one in medicine and bioethics, and the other in philosophy and bioethics, who evaluated relevance, clarity, and theoretical coherence. A pilot test was conducted with 2 second-year family medicine residents, who were not included in the final sample. The duration and narrative depth led to minimal adjustments to the instructions, leaving the final version with five open-ended main questions.

After providing digital informed consent, participants self-recorded their responses in a private setting of their choosing, with a suggested duration of 15–30 min to favor narrative depth. 28 self-conducted guided interviews were carried out and video-recorded asynchronously using a thematic script. One in-depth self-recorded interview was conducted per participant; repeated interviews were not conducted. They lasted between 9 min 57 s and 22 min 40 s, with an average of 16 min 16 s, a standard deviation of 3 min 10 s, a median of 16 min 5 s, and a total accumulated duration of 456 min of video material. Recordings were submitted electronically and reviewed by the research team. Since this is a sensitive topic with a minimal risk of emotional impact, a first-level support strategy was put in place: after receiving each recording, the principal investigator made a follow-up call and debriefing to (a) thank the participant for their involvement, (b) check in on their emotional state after the recording, and (c) offer a referral to institutional psychological support if needed. No participants requested a referral.

Videos were transcribed verbatim; field notes documented emotional reactions, nonverbal language, significant expressions, and silences to help reconstruct symbolic elements of participants’ narratives.

### 2.4. Data Analysis

Consistent with the hermeneutic phenomenological orientation, this sequence was not applied linearly; categories and interpretations were revisited iteratively as new transcripts were incorporated.

Data were analyzed using ATLAS.ti 25 following a phenomenological analytic sequence: (1) integral reading of interviews and field notes; (2) open coding of experiential meaning units, with particular attention to shared beliefs, cultural tensions, and symbolism; (3) iterative hermeneutic interpretation of these meaning units within the participants’ lived experience; (4) construction of interpretive categories; and (5) narrative synthesis integrating quotations, narratives, and observations. Interview questions were organized around five a priori analytic dimensions (Table 1).

The five a priori dimensions were not thematic categories in the sense of Braun & Clarke, but rather the operational translation of the phenomenological framework in the interview guide. Open coding within each dimension was the actual phenomenological step: identifying units of lived meaning, not recurring themes in the sense of prevalence.

Subsequently, these meaning units were subjected to iterative hermeneutic interpretation to explore the meanings participants attributed to their own mortality, future expectations, autonomy, suffering, and professional identity. The analysis moved iteratively between individual narratives and the emerging structure of meanings, consistent with an interpretive phenomenological approach. To illustrate this movement from individual narrative to interpretive category, consider participant P17’s statement: *“If diagnosed with a serious, untreatable illness, I would only want the staff caring for me to treat me with dignity, to understand the situation… and to communicate with me clearly throughout.”* Read descriptively, this excerpt is a preference statement about clinical communication. Read hermeneutically—situating it within the whole of P17’s narrative and against the horizon of the other 27 accounts (the hermeneutic circle previously described)—the excerpt discloses something further: dignity is not experienced as a property added to good clinical care, but as the condition under which suffering remains bearable and the self remains recognizable to others. This interpretation is what allowed the excerpt to be coded as “dignity” within the “desired medical care” category (Table 2), and to be read as an expression of van Manen’s lived body existential. The coding tree and categories thus did not emerge from counting recurring words or phrases, but from this repeated interpretive movement between individual excerpts and the developing structure of meaning across the sample.

Open coding of the transcripts and field notes yielded a hierarchical coding tree organized into one overarching macro-category (end of life), five categories aligned with the a priori analytic dimensions, and 25 descriptive codes capturing recurrent units of meaning within each category (Table 2). Codes were generated inductively from participants’ narratives and grouped deductively under the five categories that had informed the interview guide. Coding was performed by a single investigator; to strengthen credibility and confirmability, a second coder with graduate studies in educational research focused on qualitative and quantitative methods conducted a full plausibility audit of the resulting coding tree. The second coder did not generate an independent coding scheme; rather, for all 28 transcripts, the second coder verified that each assigned code was adequately supported by its corresponding textual excerpt, and reviewed the internal coherence of the category structure as a whole. The audit was conducted against the coding tree in its final form, rather than against a blinded or pre-coded version of the transcripts. Discrepancies concerning code-to-excerpt fit and category placement were discussed between the principal investigator and the second coder until consensus was reached.

All participants received written confirmation from the research team acknowledging their participation and confirming the accuracy of the sociodemographic and contextual data recorded for them. In addition, preliminary findings were shared with a subset of five participants, who reported finding the results interesting and raised no objections regarding the interpretation presented or the way their contributions were represented; because quotations were anonymized and no names were disclosed, no concerns regarding identifiability were reported. This partial respondent validation was not systematically extended to the full sample or to each thematic category individually, and is therefore a limited member-checking procedure.

### 2.5. Ethical Considerations

In accordance with the Mexican General Health Law Regulations on Health Research (Article 17, Section I), this study was classified as minimal-risk research, as it involved a qualitative phenomenological design using a self-administered interview guide. In accordance with the Declaration of Helsinki, the protocol was reviewed and approved by the Local Health Research Committee (CLIS 1003) and the Research Ethics Committee (CEI 10038) of HGZ MF No. 2, IMSS. Participant confidentiality was safeguarded throughout data collection, analysis, and dissemination; all participants provided informed consent and retained the right to withdraw at any time without consequences for their institutional record. Direct quotations are identified by anonymized participant codes (P1–P28) rather than by name.

### 2.6. Reflexivity

The principal investigator is a medical specialist in Anesthesiology and Bioethics with a postgraduate diploma in Palliative medicine, with Master’s degrees in Human Rights, and Critical thinking and Hermeneutics, with a postgraduate diploma in Qualitative research. He holds a clinical and teaching position within the residency training program at HGZ MF No. 2, the same institution where the study was conducted. This dual role as clinician-educator and researcher constitutes an insider position that may have shaped both data collection and interpretation. All the participants previously knew the main investigator in their clinical-teaching role and they were informed about the purpose of the study, establishing that the main goal was to encourage introspection on topics that are usually not discussed in their age group, particularly about dealing with illness and death. The asynchronous, self-recorded interview format was adopted in part to mitigate social-desirability bias arising from this relationship, allowing participants to respond without the investigator present. The academic advisor, a philosopher with no clinical role or institutional affiliation with the hospital, contributed an external perspective during analysis and manuscript preparation, providing a partial counterbalance to the main investigator’s insider position.

This reflexive account should be understood not as an attempt to eliminate the principal investigator’s clinical and educational pre-understanding, but as its explicit articulation—a distinction central to the hermeneutic phenomenological tradition adopted in this study. Norlyk and Harder (2010) note that many studies claiming a phenomenological approach fail to specify how such philosophical principles were concretely implemented, often defaulting to a generic reference to ‘bracketing’ without clarifying which variant was practiced or why (Norlyk & Harder, 2010). Consistent with Heidegger’s rejection of a presuppositionless standpoint, no attempt was made to suspend or set aside the principal investigator’s fore-structure of understanding as clinician-educator; instead, this fore-structure was made explicit here, and was treated during analysis as an interpretive resource to be examined critically rather than as contamination to be controlled. Concretely, this meant that whenever an interpretive claim drew on the principal investigator’s clinical familiarity with participants’ professional context, that claim was discussed with the academic advisor—who, lacking clinical affiliation with the hospital, could identify where the principal investigator’s institutional closeness might be shaping the reading of a transcript—and was revised or retained accordingly. This procedure is what operationalized, in practice, the hermeneutic circle described in Section 2.1.

To reduce coercion and social desirability due to hierarchical asymmetry, written informed consent was obtained that made it clear that participation was voluntary and without any extra care burden, and also stated their right to withdraw from participation without needing to justify leaving. Participants were explicitly informed that participation or non-participation would have no influence on residency evaluations, academic standing or institutional relationships. The asynchronous interview format was also intended to minimize the influence of the investigator’s dual role as educator and researcher.

### 2.7. Trustworthiness

Trustworthiness was addressed according to the criteria of credibility, dependability, confirmability, and transferability. Credibility was enhanced through verbatim transcription, maintenance of field notes, independent review of the coding structure, and respondent validation with a subset of participants. Dependability was supported through documentation of analytic decisions during coding and category development. Confirmability was strengthened through reflexive discussions between the principal investigator and the academic advisor, who provided an external perspective during interpretation. Transferability was facilitated through detailed description of the institutional setting, participant characteristics, and research procedures.

## 3. Results

### 3.1. Sociodemographic Characteristics

The specialty distribution presented in Table 3 corresponds to the initially recruited cohort of 28 residents which were analyzed. The final analytic sample retained representation from all participating specialties.

### 3.2. General Perceptions of the End of Life

Participants’ accounts of the end of life were multifaceted, shaped jointly by clinical experience, personal values, and sociocultural background. For most, this was not the first time they had considered or discussed these issues with family or friends; roughly one third, however—seven women and two men—reported never having previously considered these questions, or actively avoiding them.

During the interviews, a serious, formal tone frequently gave way to visible anguish, momentary uncertainty, or sadness, particularly when discussing imagined family loss or euthanasia; eight participants showed signs of being moved when entering the introspective process of the question, some with a broken voice, others even with teary eyes, often upon recalling a recent family illness or realizing the eventual loss of their parents.

Two dimensions emerged in tension. The first was an idealization of the end of life: participants generally envisioned dying at an advanced age, in a familiar environment, surrounded by a partner and loved ones, retaining autonomy and quality of life, with health remaining essentially intact until the final days—a romanticized, almost literary “successful ending” in which dignity is inherent to the narrative protagonist:

*“I imagine I will live many years, that I’ll become an old woman of ninety-something, and at the end of my life, I picture myself with family, with children, with grandchildren, already retired… with the most common illnesses but, well, no terminal illness…”*

(P1)

Taken together, these narratives suggest that residents inhabit a transitional space between a culture of cure and an acceptance of mortality. While they continue to identify strongly with medicine’s life-preserving mission, repeated exposure to suffering appears to foster a parallel appreciation for dignity, limitation, and the meaningful completion of life.

The second dimension was the reality of clinical experience: daily confrontation with suffering, death, and at times imprudent prolongation of life, which produced a tension between the classical medical ethic of “prolonging life” and an emerging ethic centered on “preserving quality of life.” Some participants explicitly described this topic as uncomfortable or avoided it altogether:

*“This topic is conflicting for me because I don’t think anyone at our stage of life is really thinking about their own death… illness and death give no warning, they are sudden and unexpected, and they inflict pain on the person who suffers them and on those who witness it, and can generate hopelessness and guilt.”*

(P21)

*“I honestly don’t like thinking that far ahead, I don’t do it because there are too many variables I can’t control.”*

(P25)

### 3.3. Professional, Emotional, and Family Expectations

Nearly all participants described medicine as a life vocation, while simultaneously acknowledging its emotional toll; most envisioned themselves as fulfilled, respected physicians in retirement. A majority (24 residents) expressed concern about work–life balance and resistance to a life wholly devoted to work without framing this as a limit on professional commitment, but rather as a search for equilibrium. Several expressed interest in combining clinical practice with teaching, research, or activities outside the health field.

Reflection on interpersonal relationships elicited the strongest emotional expression. Most participants considered forming a family central to their life plans, and nearly all identified partnership as an ideal, frequently attributing to this imagined future partner—rather than to current relationships—the responsibility for safeguarding their medical wishes at the end of life. A small subgroup, four male participants, expressed a preference for reaching the end of life without descendants, prioritizing professional dedication, though without ruling out a future stable partnership.

*“I consider that this [the end of life] will be alongside the family I build along the way—whether a spouse, children, as well as blood relatives who have already been with me, and their own families, and the friendships that have endured throughout this journey.”*

(P4)

*“I would like to practice private medicine, set a schedule, 8 a.m. to 3 p.m., not enslave myself or work at two or three institutions at once, to have a quiet life even without luxuries… I don’t want to be a slave to my work.”*

(P23)

### 3.4. Decisions Regarding Terminal Illness

Confronting the hypothetical scenario of terminal illness placed participants before their own fragility and finitude. Some viewed death as a natural process to be respected, at times integrating personal religious beliefs; the large majority, however, emphasized the need for medical options that avoid suffering, such as access to palliative care or assisted dying in cases of prolonged agony. Participants uniformly stated they would prefer palliative management over invasive treatment if diagnosed with a terminal illness and a preference for dying at home, surrounded by loved ones, preserving quality of life and family closeness in the final days. Several recounted distressing experiences with patients subjected to imprudent therapeutic efforts.

Regarding surrogate decision-making, most participants named a first-degree relative—most commonly their mother—followed by an imagined future partner and, in third place, future children.

*“Sometimes prolonging life causes more suffering than benefit, without real quality of life; if I were over 90 with no treatment left, no hope of recovery, I wouldn’t want to keep fighting—as long as I’m not suffering, as long as there’s no pain, I would be at peace with that.”*

(P1)

*“If diagnosed with a serious, untreatable illness, I would only want the staff caring for me to treat me with dignity, to understand the situation… and to communicate with me clearly throughout.”*

(P17)

*“I would like to receive care at home if possible… being a patient—or a family member of one—in a hospital is very hard… if I could, I would rather be cared for at home.”*

(P3)

### 3.5. Opinions on Euthanasia

Reflection on euthanasia elicited the second-greatest emotional response among participants, frequently anchored in the recollection of a loved one’s prolonged, poorly controlled suffering; several participants stated that, had euthanasia been available, they would have requested it on that person’s behalf. Three broad positions emerged.

Some participants (16/28 residents) supported euthanasia as an individual right, arguing that extreme suffering justifies medical intervention to hasten death and that such decisions cannot be judged from outside the lived experience of suffering:

*“In our country very little is said about euthanasia… but I don’t have an opinion against it; I think it is a very personal decision worth making when the situation warrants it—in terminal illnesses beyond medical capacity, where there is no possibility of cure. I consider it a valid, explorable option, and if I were ever in that situation myself, it would be an important decision worth making.”*

(P10)

A second group (7/28 residents) held an intermediate position, accepting euthanasia only under strict regulation and psychological–medical protocolization justifying the intervention. A third group (5/28 residents) was partially opposed, rejecting euthanasia for religious or professional reasons for themselves while allowing that it might be considered for others facing extreme suffering:

*“I wouldn’t do it, definitely not, because I have this thing about not wanting anything to do with someone’s death, even for their own good… I feel God is the one who gave them life, and only God can decide.”*

(P22)

*“Just recently I had a very close case… and in the end, when he passed, all we could say was: thank you—because he was already suffering so much… I used to say that if euthanasia were allowed, we would have done it earlier.”*

(P8)

### 3.6. Advance Directives

Participants recognized advance directives as a direct expression of autonomy. Although a large majority said they had talked about it with their families and friends, none of the participants had made a formal advance directive at the time of the study.

Elements they would include were: organ donation; refusal of invasive treatment if the prognosis is irreversible; prioritization of palliative over life-sustaining care; a preference for dying at home surrounded by loved ones; delegation of decision-making to a trusted family member; and, for some, the option of euthanasia in the event of intractable suffering.

*“There are particular things I would like to leave to certain friends, to my partner, to my parents… whatever they want, they keep, and the rest they can give away or discard—that would be their choice… in the end, what stays with people is who you were in life, more than material things, which only help with memory. I’ve thought about what should happen to my body; I’ve always liked the idea of those sacred forests, where they plant many trees and lay people to rest there—I find it a beautiful idea.”*

(P2)

*“I’ve already told my family, mainly my mother, since she’s the one I see as most capable of understanding these things—if something happens to me, if I end up in a coma, in a vegetative state so to speak… she already knows: donate my organs.”*

(P19)

Figure 1 is a word cloud that shows the predominance of words reflecting an idealis-tic vision of the future contrasted with concepts of adversity that emerge and become sig-nificant. The image is just an exploratory supplementary illustration and not primary analytical evidence.

## 4. Discussion

Findings show that clinical experience does not immunize physicians against the fears and dilemmas surrounding death; rather, frequent contact with suffering may be associated with a pragmatic orientation in which quality of life outweighs its artificial prolongation. These findings resonate with recent qualitative work. Harhara et al. (2022) similarly found that internal medicine residents undergo a process of emotional confrontation, coping, and meaning-making when exposed to patients in palliative care, describing professional growth alongside distress and a more pragmatic reorientation toward quality of life (Harhara et al., 2022).

A recurring theme was the idealization of death as painless, conscious, and accompanied—consistent with what Han describes as the “palliative society,” in which “algophobia is ultimately thanatophobia” (Han, 2020/2021, p. 29), leaving little space for suffering as a source of meaning or growth, despite the inescapable fragility of life. This idealization can be read less as denial than as an inarticulate confrontation with mortality: even when death itself remains uncertain, pain is universally recognized and understood, which may explain why avoiding it becomes the more tangible object of concern.

From a phenomenological perspective, participants did not describe death merely as a biological outcome but as an existential horizon through which personal relationships, professional aspirations, autonomy, and suffering acquired meaning. Their narratives reveal a continuous negotiation between the identity of the physician and the awareness of personal finitude.

The consistent expression of concern for work–life balance—rather than unconditional dedication to the profession—echoes a broader generational shift documented among health-care students and professionals internationally, with younger cohorts increasingly prioritizing personal well-being alongside professional demands (Kanste et al., 2025).

When contrasting their idealized future with a hypothetical terminal illness, participants consistently favored palliative over invasive measures, applying, implicitly, the bioethical principle of therapeutic proportionality; this orientation appeared strongly shaped by prior exposure—whether clinical or personal—to the suffering of others, which also informed responses regarding advance directives and euthanasia. On that matter, Jedlicska et al. (2024) found that specific formative encounters with death and dying during training left lasting imprints on trainees’ broader professional and personal attitudes, rather than being processed as isolated clinical events (Jedlicska et al., 2024).

The emotional overflow observed in several interviews is itself a significant finding. It suggests that reflection on personal mortality, when facilitated in a safe space, activates deep emotional resonances tied to prior grief and anticipated future loss—resonances that within this sample neither medical schools nor hospitals, as institutions, provided space to process, leaving residents to manage them privately or suppress them altogether. This is consistent with prior findings that end-of-life experiences, when appropriately supported, foster critical perspectives on values, priorities, and professional meaning among physicians-in-training (Strano-Paul et al., 2017). That participants can articulate, once given the opportunity, a clear preference for dignity-preserving, proportionate care—while practicing within a system that still frequently defaults to therapeutic obstinacy—reflects their position early in professional socialization within a hospital apparatus still oriented toward curing and avoiding death, rather than a contradiction in their values.

Even though the residents participating in the study had already completed the basic training curriculum of the medical degree and even the module corresponding to medical ethics, their preparation to address end-of-life issues, even when treated as a reflection exercise, still causes difficulty and emotional distress. Lisai-Goldstein and Shaulov’s (2025) review of how medical trainees process a patient’s death reported that despite institutional time and resources devoted to preparing students in that subject, many still feel inadequately prepared when it occurs. Strategies that make it easier for residents to approach issues that are rarely addressed in clinical practice, such as a patient’s death, delivering bad news, or coping mechanisms to deal with emotional stress and burnout, should be part of the hospital training routine (Lisai-Goldstein & Shaulov, 2025). Narrative medicine, which recovers individual and collective narratives as a pedagogical tool, may offer a promising avenue for addressing these training gaps and warrants further consideration in academic curricula (Loy & Kowalsky, 2024).

The finding that an imagined future partner—rather than existing relationships—was frequently prioritized in the decision-making hierarchy is notable from a phenomenological standpoint: it suggests that residents construct prospective narratives in which partnership functions as an anchor for future autonomy, with potential implications for how advance-directive documents are designed and discussed.

Regarding euthanasia, the diversity of positions found, and the prominence of personal grief as a catalyst for reflection, suggests that this topic cannot be addressed in training solely as an abstract matter of principle; the lived, experiential dimension of euthanasia as an unused possibility in the face of a loved one’s suffering is a central narrative element that bioethics curricula should engage directly. This is consistent with prior work showing that clinicians with greater exposure to therapeutic proportionality and palliative care tend to be more attuned to patient wishes and more open to considering euthanasia as a distinct option alongside palliative care, even as the topic remains ethically contested and can create tension between personal conviction and clinical knowledge (Campos Gutiérrez et al., 2024; Dedivitis et al., 2023; Mimarakis et al., 2025).

Finally, the gap between low rates of formal advance-directive completion—both nationally and within this cohort prior to the study—and the clarity with which participants could articulate their wishes once given a structured space for reflection suggests that the barrier is not an absence of opinion but a lack of structured opportunities for its expression and formalization.

Despite the declared limitation, the professor-resident relationship became a subject of reflective analysis: the fact that the residents recorded themselves answering a guided interview allowed access to more vulnerable expectations that might not have been expressed in a traditional interview in front of a professor or a researcher.

### Limitations

The study has limitations that need to be pointed out. (a) The self-recorded, asynchronous interview format, while reducing social-desirability bias toward a physician-investigator, precluded real-time probing and the observation of nonverbal communication during the interview itself. Consequently, some experiential dimensions may not have been explored with the depth achievable through face-to-face phenomenological interviewing. (b) There is a potential influence of the principal investigator’s dual clinical-teaching position, despite the mitigation measures described in Section 2.6. (c) The study was conducted at a single institution with a purposive, census-type sample of residents from a single, curricularly delimited cohort, which by design offers depth within a specific and homogeneous group, limiting transferability to other training stages, specialties, or institutional cultures. (d) All participants had prior ethics training, limiting any inference about training effects. (e) Member-checking was conducted with a limited subset of participants (*n* = 5). (f) Although the analysis was done in Spanish, a possible loss of nuances with the translation into English is acknowledged.

As with all qualitative phenomenological research, findings are intended to offer an in-depth understanding of meaning within this specific group.

## 5. Conclusions

This qualitative phenomenological study examined the narrative representations and end-of-life expectations of first-year medical residents at a Mexican general hospital, encompassing their professional, family, and social expectations, their preferences regarding treatment in the event of serious or terminal illness, and their views on euthanasia and advance directives. Findings show that residents hold a nuanced view of death, shaped jointly by clinical experience, personal values, and sociocultural background. Although most favor autonomy and palliative care, unresolved tensions persist around euthanasia and surrogate decision-making, reflecting a generation navigating between the traditional biomedical model and a more humanistic medicine.

Increasing curricular exposure to medical ethics and palliative care appears to be fostering a positive shift away from a purely biomedical model; however, this academic exposure does not consistently translate into spaces where residents can emotionally process their own relationship with death and suffering. Central findings include the construction of idealized end-of-life narratives shaped by family and sociocultural influence, alongside more pragmatic, quality-of-life-oriented representations shaped by direct clinical exposure to suffering and death. The emotional responses observed during several interviews suggest the possible existence of unmet needs for emotional support among residents, a finding that warrants further exploration in future studies. Most participants would prefer to die at home with palliative care rather than undergo invasive treatment—a preference that contrasts with prevailing hospital practice in Latin American countries—and while advance directives were positively valued, their formalization remains exceptional both in the general population and among physicians themselves. The euthanasia debate, in particular, is inseparable from participants’ personal experiences of grief, which complicate any purely theoretical approach to the topic.

The findings suggest that first-year residents occupy a liminal position between the traditional biomedical paradigm and a dignity-centered approach to care. Reflection on personal mortality may contribute to the development of more humane and person-centered professional perspectives.

Taken together, these findings underscore the need to integrate reflection on death into medical training—not only as a technical matter but as a human and ethical one. Incorporating structured reflection on finitude into specialist training is not an academic luxury; it is a necessary condition for building a medical culture equal to the human dignity it is meant to serve.

## Figures and Tables

**Figure 1:**
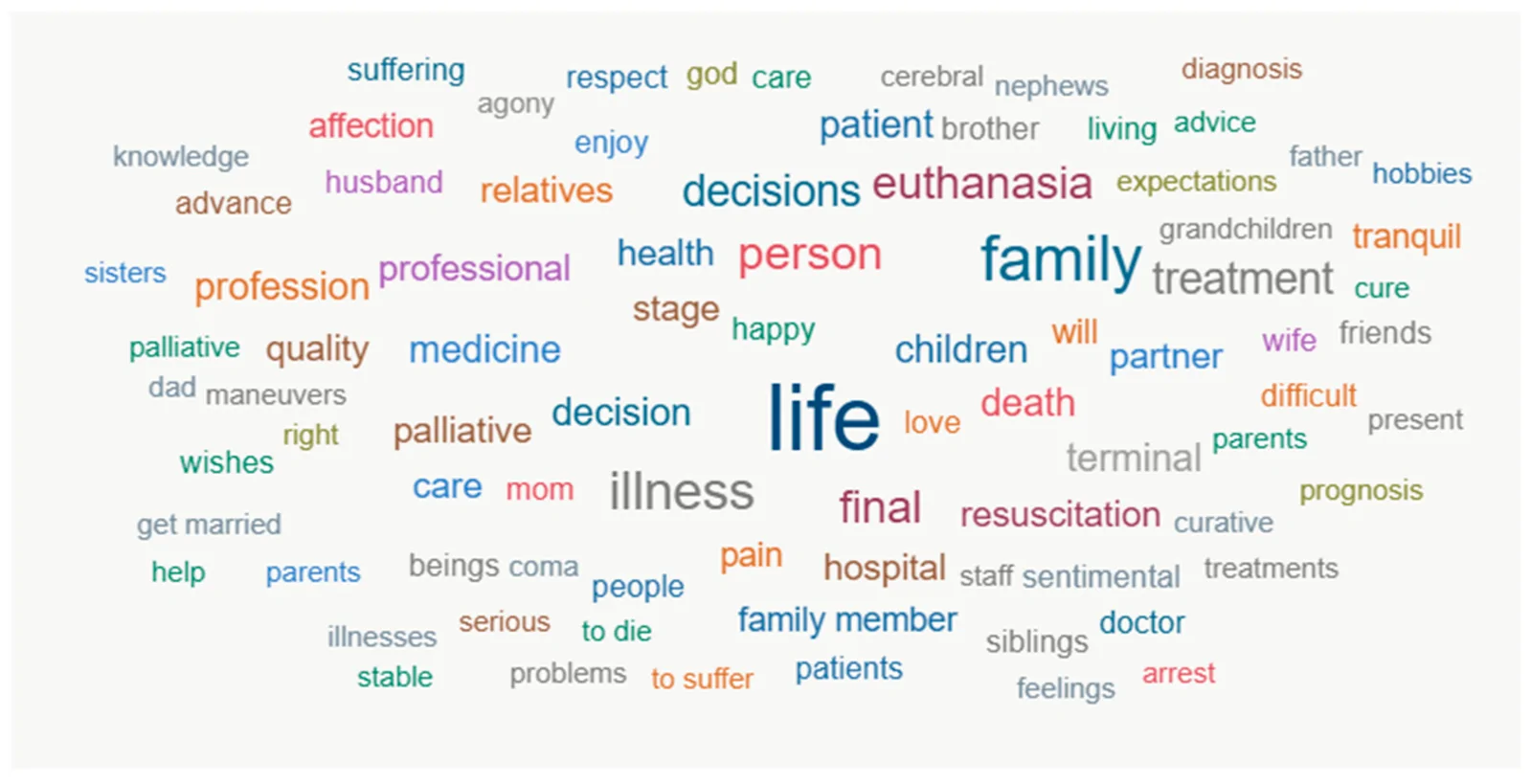
Word cloud of the interviewees. Author-generated using ATLAS.ti. A larger font size for the words represents a higher frequency of occurrence in the participants’ testimonies.

**Table 1:** A priori analytic dimensions guiding the interview guide.

Dimension	Operational Description
Representation of the end of life	How residents imagine or conceptualize their own terminal stage
Relational expectations	Desires and projections regarding family, emotional, and professional bonds at the end of life
Desired medical care	Preferences regarding the type, place, and conditions of care (hospital, home, palliative care)
Advance directives	Attitudes toward advance directives, prior decisions, and self-determination
Position on euthanasia	Personal and professional opinion on ending life under consent

**Table 2:** Hierarchical coding tree derived from open coding. Categories deductively derived from the theoretical framework/interview guide; codes inductively derived from the data within each category.

Macrocategory	Category	Codes
End of life	Representation of the end of life	Tranquil idealized ending; uncertainty; avoidance
	Relational and professional expectations	Family; descendants; partner; pets; work–life balance; professional fulfillment; legacy; recognition
	Desired medical care	Suffering avoidance; dignity; palliative care; home setting; surrogate decision-maker
	Advance directives	Content/wishes; organ donation; do-not-resuscitate
	Opinion on euthanasia	Others’ suffering; ethical/religious conflict; in favor; against; personal consideration; not for me

**Table 3:** Sociodemographic characteristics of the recruited sample.

Characteristic	Value
Residents recruited	28
Women	17
Men	11
Age range (years)	25–33
Family medicine residents	4
Anesthesiology residents	3
Surgery residents	8
Internal medicine residents	9
Pediatrics residents	4

## Data Availability

The interview transcripts analyzed in this study are not publicly available due to the sensitive nature of qualitative data concerning personal reflections on death and the ethical commitment to participant confidentiality made at the time of consent. De-identified excerpts may be made available by the corresponding author upon reasonable request and subject to Research Ethics Committee approval.

## Abbreviations

The following abbreviations are used in this manuscript:

IMSS	Instituto Mexicano del Seguro Social
HGZMF	Hospital General de Zona con Medicina Familiar
CLIS	Comité Local de Investigación en Salud
CEI	Comité de Ética en Investigación
COREQ	Consolidated Criteria for Reporting Qualitative Research
WHO	World Health Organization

## Appendix A. Interview Guide

Guiding considerations were offered only as optional prompts to support reflection, not as directive sub-questions; participants were free to respond to the open-ended question without addressing them.

**Table A1:** Interview Guide.

Dimension	Open Question	Guiding Considerations
Representation of the end of life	ES: ¿Cómo me imagino que será mi etapa final de vida? EN: How do I imagine my end-of-life stage will be?	ES: Considera expresar tu respuesta a detalle. EN: Consider expressing your answer in detail.
Relational and professional expectations	ES: En mi etapa final de vida, ¿cuáles serían mis expectativas con respecto a lo familiar, sentimental y profesional? EN: In my end-of-life stage, what would my expectations bevregarding family, emotional, and professional matters?	ES: Considera si te visualizas con una vida que sea producto de la práctica médica. EN: Consider whether you picture a life shaped by your medical practice.
Desired medical care	ES: En caso de ser diagnosticado con alguna enfermedad grave o terminal fuera de tratamiento curativo, ¿cuáles serían tus expectativas respecto a tu atención médica? EN: If diagnosed with a serious or terminal illness beyond curative treatment, what would your expectations be regarding your medical care?	ES: Considera a quién dejarías a cargo de las decisiones importantes respecto a tu salud en caso de no poder expresar tu voluntad. Considera si preferirías mantener tu atención en casa o en domicilio. EN: Consider whom you would designate to make important health decisions if unable to express your wishes, and whether you would prefer care at home.
Advance directives	ES: Si tuviera que hacer una voluntad anticipada ahora, ¿qué incluiría? EN: If you had to draft an advance directive now, what would you include?	ES: Considera deseos de medicina paliativa contra la medicina convencional, quién tomaría las decisiones médicas importantes, situaciones en las que no quisieras ser sometido a reanimación, etc. EN: Consider preferences for palliative versus conventional care, who would make key medical decisions, and situations in which you would not want resuscitation.
Position on euthanasia	ES: ¿Qué opinas de la eutanasia? EN: What is your opinion on euthanasia?	ES: Considera si en algún momento la considerarías para ti o para algún ser querido. EN: Consider if you would you ever consider it for yourself or for a significant other.
